# Determination of traits responding to iron toxicity stress at different stages and genome-wide association analysis for iron toxicity tolerance in rice (*Oryza sativa* L.)

**DOI:** 10.3389/fpls.2022.994560

**Published:** 2022-10-06

**Authors:** Cattarin Theerawitaya, Samart Wanchana, Vinitchan Ruanjaichon, Rujira Tisaram, Thapanee Samphumphuang, Thanyaporn Sotesaritkul, Suriyan Cha-um, Theerayut Toojinda

**Affiliations:** National Center for Genetic Engineering and Biotechnology (BIOTEC), National Science and Technology Development Agency (NSTDA), Khlong Luang, Pathum Thani, Thailand

**Keywords:** genome-wide association studies, iron toxicity, QTL, reproductive stage, rice (*Oryza sativa*), seedling stage, vegetative stage

## Abstract

Rice is the staple food for more than half of the world’s population. Iron toxicity limits rice production in several regions of the world. Breeding Fe-tolerant rice varieties is an excellent approach to address the problem of Fe toxicity. Rice responds differently to Fe toxicity at different stages. Most QTLs associated with Fe toxicity have been identified at the seedling stage, and there are very few studies on Fe toxicity across different stages. In this study, we investigated agro-morphological and physiological traits in response to Fe toxicity in a rice diversity panel at seedling, vegetative, and reproductive stages and applied GWAS to identify QTLs/genes associated with these traits. Among agro-morphological and physiological parameters, leaf bronzing score (LBS) is a key parameter for determining Fe toxicity response at all stages, and SDW could be a promising parameter at the seedling stage. A total of 29 QTLs were identified on ten chromosomes. Among them, three colocalized QTLs were identified on chromosome 5, 6, and 11. Several QTLs identified in this study overlapped with previously identified QTLs from bi-parental QTL mapping and association mapping. Two genes previously reported to be associated with iron homeostasis were identified, i.e., LOC_Os01g72370 (*OsIRO2, OsbHLH056*) and LOC_Os04g38570 (*OsABCB14*). In addition, based on gene-based haplotype analysis, LOC_Os05g16670 was identified as a candidate gene for the colocalized QTL on chromosome 5 and LOC_Os11g18320 was identified as a candidate gene for the colocalized QTL on chromosome 11. The QTLs and candidate genes identified in this study could be useful for rice breeding programs for Fe toxicity tolerance.

## Introduction

Iron (Fe) is a crucial micronutrient for normal growth in most living organisms. Fe plays a key role as a chelating molecule in plant metabolic activities, such as mitochondrial respiration, photosynthesis, electron transport, and other redox reactions (Kobayashi and Nishizawa, 2012; Li and Lan, 2017; Aung et al., 2018). However, excessive Fe accumulation in cells can cause Fe toxicity, leading to nutritional disorders, physiological and agronomic depletion, and even plant death (Kobayashi and Nishizawa, 2012; Sperotto et al., 2012; Mahender et al., 2019; Rasheed et al., 2020). Fe toxicity is a serious constraint among abiotic stresses on rice productivity in several areas of the world. About 18% of soils worldwide suffer from Fe toxicity (Mahender et al., 2019).

Among the essential carbohydrate sources that feed the world’s population, rice is the most important crop, providing over 21% of the caloric needs of the entire world population and 76% of the needs in Southeast Asia (Fitzgerald et al., 2009). Reductions in rice production in lowland areas under Fe stress ranged from 12 to 100%, depending on rice genotype, Fe contaminated level, and soil fertility (Audebert and Fofana, 2009; Sahrawat, 2010; Engel et al., 2012). The availability of Fe toxicity tend to increase by the conversion of Fe^3+^ to the reduced ferrous form of Fe^2+^ under waterlogged acidic soils, anaerobic condition found in tropical lowland rice fields (Rasheed et al., 2020). The excess Fe^2+^ is transported from the root to the shoot and leads to leaf discoloration (bronzing), cellular oxidative damage, nutrient deficiency, and stunted growth of rice (Sikirou et al., 2016; Wu et al., 2017; Turhadi et al., 2019; Tisarum et al., 2022). Among the physiological and agronomic responses to Fe toxicity, several genes have been involved in multiple responses, including Fe uptake, translocation, subcellular Fe translocation, and Fe regulation (Kobayashi and Nishizawa, 2012; Rasheed et al., 2020). Breeding Fe-tolerant rice varieties is an excellent approach to address the problem of Fe toxicity. Thus, a marker-assisted breeding program is a promising strategy to breed rice varieties with Fe tolerance. There are several attempts to identify quantitative trait loci or QTLs/genes associated with Fe toxicity using intra-specific and inter-specific mapping populations in rice (Wan et al., 2003; Ouyang et al., 2007; Shimizu, 2009; Dufey et al., 2015; Diop et al., 2020). From 244 RILs of Zhenshan 97B/Miyang 46, seven QTLs have been found on chromosomes 1, 4, 5, and 7, with LOD scores ranging from 2.88 to 15.94, associated with the effect of stimulating coleoptile elongation rate (Ouyang et al., 2007). Twenty-eight QTLs in 18 different chromosomal regions associated with morphological and physiological traits have been obtained from a population of 220 BC_3_DH of *O. sativa* (Caiapo)/*O. glaberrima* (MG12)/*O. sativa* (Caiapo) tested under hydroponic conditions for Fe^2+^ 250 mg L^−1^ (Dufey et al., 2015). Genome-wide association studies (GWAS) have also been applied to identify QTLs/genes associated with Fe toxicity-related traits in rice (Matthus et al., 2015; Zhang et al., 2017; Diop et al., 2020; Melandri et al., 2020; Kaewcheenchai et al., 2021).

Defensive strategies of rice under Fe toxicity at different developmental stages are differently attempted (Sikirou et al., 2016). Most QTLs associated with Fe toxicity have been studied at the seedling stage under hydroponic conditions, with few at the reproductive stage (Diop et al., 2020; Melandri et al., 2020). There are very few literatures describing QTLs associated with traits responding to Fe toxicity at seedling, vegetative, and reproductive stages. In this study, we evaluated agro-morphological and physiological traits in response to Fe toxicity at seedling, vegetative, and reproductive stages in a rice diversity panel, mainly indica rice from Thailand, and selected appropriate traits to identify QTLs/genes associated with iron toxicity tolerance using GWAS.

## Materials and methods

### Plant materials and SNP genotyping data

Total of 239 rice accessions including 119 Thai landraces, 82 improved genotypes, and 38 international varieties were used in this study (Supplementary Table S1). Single nucleotide polymorphism (SNP) genotype data for the 239 accessions came from a whole-genome resequencing project conducted by the Plant Biotechnology and Precision Agriculture Research Team, National Center for Genetic Engineering and Biotechnology, Thailand (unpublished data), and were called using the Nipponbare IRGSP 1.0 rice reference genome. SNPs with a miss rate greater than 30% and minor allele frequency (MAF) less than 5% were removed. Heterozygous alleles were also excluded. Finally, 160,498 SNPs were selected and used in the GWAS analysis (Supplementary Table S2; Supplementary Figure S1).

### Phenotypic analysis

The experiment was conducted in a greenhouse at Thailand Science Park, Khlong Luang, Pathum Thani, Thailand. Relative humidity and temperature were controlled at 80 ± 5% and 32 ± 2°C at the daytime/28 ± 2°C at the nighttime, respectively. Seeds of 239 rice accessions were divided into three groups for phenotypic evaluation experiment at seedling, vegetative, and reproductive stages. For the seedling stage, four replicates of rice seeds were germinated on a moist paper towel in Petri dishes. After 3 days, the germinated seeds were sown in a commercial soil until a few leaves appeared. Rice seedlings were then transferred individually into plastic plates floating in a plastic container filled with distilled water. The 14-day-old rice seedlings were then transferred to water containing 0 or 100 mM FeSO_4_ solution, pH 4.5, as provided for control and iron toxicity, respectively. For vegetative and reproductive stages, rice seeds were sown with four replicates per accession and grown in commercial soil with chemical properties of electrical conductivity = 2.7 dS m^−1^, pH = 5.7, total organic carbon = 12.3%, available *N* = 0.3 mg kg^−1^, available *p* = 578 mg kg^−1^, available *K* = 3,073 mg kg^−1^, available Ca = 7,020 mg kg^−1^, available Mg = 1,034 mg kg^−1^. The 14-day-old seedlings were then planted in the plastic tray consisted of 80 holes with a diameter of *3.5* cm and a depth of *4.5* cm. The plastic trays were placed in a rubber tank (80 × 80 × 20 cm of width × length × height), and tap water was maintained at the soil surface. The 16-16-16 (N-P-K) fertilizer (5 g per tank) was added at 14 and 45 days after transplanting. At the vegetative stage of the plant (60 days after sowing), 0 or 100 mM FeSO_4_ solution with pH 4.5 was added to the tank of the first set of rice plants. On the other hand, the second set of rice plants was grown continuously until the booting stage. One hundred and sixty-nine accessions from the entire rice panel that were capable of flowering were added with FeSO_4_ solution of 0 or 100 mM, pH 4.5, before phenotypic traits were collected.

Agro-morphological shoot traits, i.e., shoot height (SH), shoot fresh weight (SFW), shoot dry weight (SDW), and root traits, i.e., root fresh weight (RFW) and root dry weight (RDW), were measured 14 days after treatment for the seedling stage, whereas these traits were measured 7 days after treatment for the vegetative and reproductive stages. All traits were measured and recorded according to IRRI Standard Evaluation System for rice (SES, 2013). SH was measured from the base of rice plants to the tip of the uppermost leaf. SDW and RDW were measured on the harvested shoots and roots after 72-h drying in a hot air oven at 80°C. Physiological traits, i.e., maximum quantum yield (F_v_/F_m_), photon yield of PSII (Φ_PSII_), leaf greenness (Soil Plant Analysis Development or SPAD value), and leaf bronzing score (LBS) were measured. Chlorophyll fluorescence emission was measured from the adaxial surface of second-fully expanded leaf at the vegetative stage or flag leaf at the reproductive stage using a fluorescence monitoring system (model FMS 2; Hansatech Instruments Ltd., Norfolk, United Kingdom) according to Loggini et al. (1999) and Maxwell and Johnson (2000). Leaf greenness was measured using SPAD (SPAD-520Plus, Konica Minolta, Osaka Japan) at the same leaf position (Hussain et al., 2000). LBS was visually scored on a scale of 1 (no symptom on leaf) to 9 (dead leaf) according to the IRRI Standard Evaluation System for rice (SES, 2013; Supplementary Figure S2).

### Population study and linkage disequilibrium analysis

The population structure of the 239 rice accessions was analyzed by principal component analysis (PCA) and phylogenetic tree using GAPIT version 3 (Wang and Zhang, 2021). Relatedness (kinship) among individuals was also estimated using the same tool. PopLDdecay (Zhang et al., 2019) was used to calculate pairwise markers LD as squared allele frequency correlation (*r*^2^) for intra-chromosomes spanning 12 rice chromosomes using a sliding window. The decay distance was determined when the average LD *r*^2^ falls to half its highest value.

### QTLs and candidate gene identification by GWAS and haplotype analyses

The phenotypic data on different morpho-physiological traits under Fe toxicity were subjected to GWAS analysis. The traits that showed a significant difference between the control (pH 4.5) and the treatments (pH 4.5 + Fe) were selected to perform GWAS using Fixed and Random Model Circulation Probability Unification (FarmCPU) in GAPIT version 3. The first three PCs and the kinship matrix were considered to reduce the occurrence of false positive and spurious associations. Quantile-quantile plots of the estimated and observed *p* values for the marker-trait association was created to evaluate model fit. The critical *p* value for explaining a significantly associated marker was the Bonferroni multiple test correction at −log10(*p*) = 6.50. Considering the LD decay distance, we defined the QTL region as the interval of significant SNPs ± LD decay distance. Based on the estimated LD (*r*^2^ = 0.3), two or more significant SNPs in the <200-kb interval were considered as a single QTL. LDBlockShow software was used to analyze LD structures and haplotype block analysis (Dong et al., 2021). The Rice Genome Annotation Project Database was used to annotate candidate genes within haplotype blocks (Kawahara et al., 2013). Candidate genes were selected based on their biologically related functions. Gene-based haplotype analysis was also performed using non-synonymous SNPs within coding sequence regions (CDSs) for candidate genes in important QTLs. Promising candidate genes were identified by testing for significant differences of the mean trait value between major haplotypes in each gene using one-way analysis of variance. Independent sample t-test was performed to determined significance of difference (5% significant level) using SPSS software version 11.5. Duncan’s test for comparison of multiple means was performed to determine the significance of differences (5% significance level).

## Results

### Variations in phenotypic traits in response to iron toxicity at seedling, vegetative, and reproductive stages

**Figure 1:**
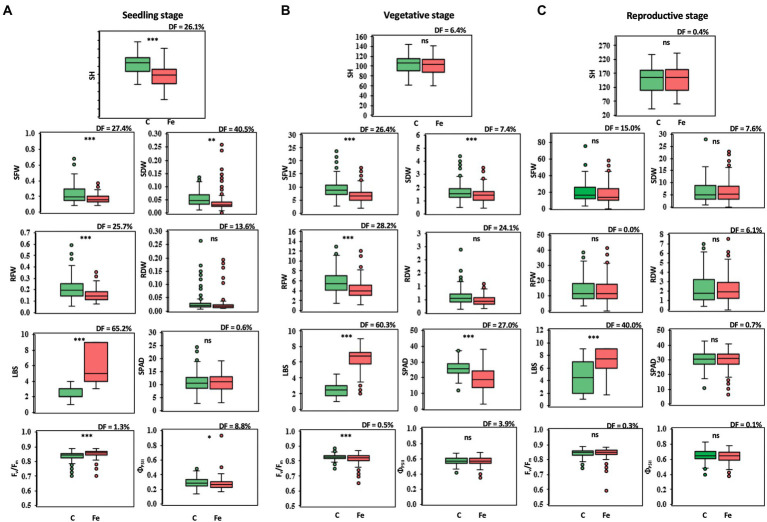
Trait variations at the seedling **(A)**, vegetative **(B)**, and reproductive stages **(C)**, comparing control (C) and iron toxicity (Fe) conditions. SH, shoot height (cm); SFW, shoot fresh weight (g plant^−1^); SDW, shoot dry weight (g plant^−1^); RFW, root fresh weight (g plant^−1^); RDW, root dry weight (g plant^−1^); LBS, leaf bronzing score; SPAD, leaf greenness index; F_v_/F_m_, maximum quantum yield of PSII; Φ_PSII_, photon yield of PSII. *, **, *** and ns mean data of C and Fe are significant different at *p* < 0.05, 0.01, 0.001 and non-significant, respectively.

Differences in phenotypic traits, both agro-morphological and physiological, were observed in 239 rice accessions at the seedling, vegetative, and reproductive stages in comparison between the control (pH 4.5) and iron toxicity (100 mM FeSO_4_ solution, pH 4.5: Fe + pH 4.5). At the seedling stage, phenotypic effects were significantly different for all variables except SPAD and RDW (Figure 1A). LBS showed the largest median difference (DF) of 65.2%. At the vegetative stage, SFW, RFW, and RDW were reduced by 26.4, 28.2, and 24.1%, respectively, when the rice culture was adjusted to iron toxicity for 7 days (Figure 1B). The adverse effect of FeSO_4_ was also evident in physiological traits; a higher degree of LBS with 60.3% increase and a reduction in greenness (SPAD) with 27.0% DF were observed. F_v_/F_m_ and Φ_PSII_ were slightly decreased after 7 days of treatment with 0.5 and 3.9%, respectively (Figure 1B). A smaller decrease or increase in some parameters was observed after 7 days of FeSO_4_ treatment at the reproductive stage (Figure 1C). For agro-morphological traits, SFW of rice under 100 mM FeSO_4_ was 15.0% lower than SFW of rice under control conditions. A lower DF level of LBS was observed at the reproductive stage (40.0%) compared to the seedling and vegetative stages (Figure 1C).

### Correlation of the different traits

**Figure 2:**
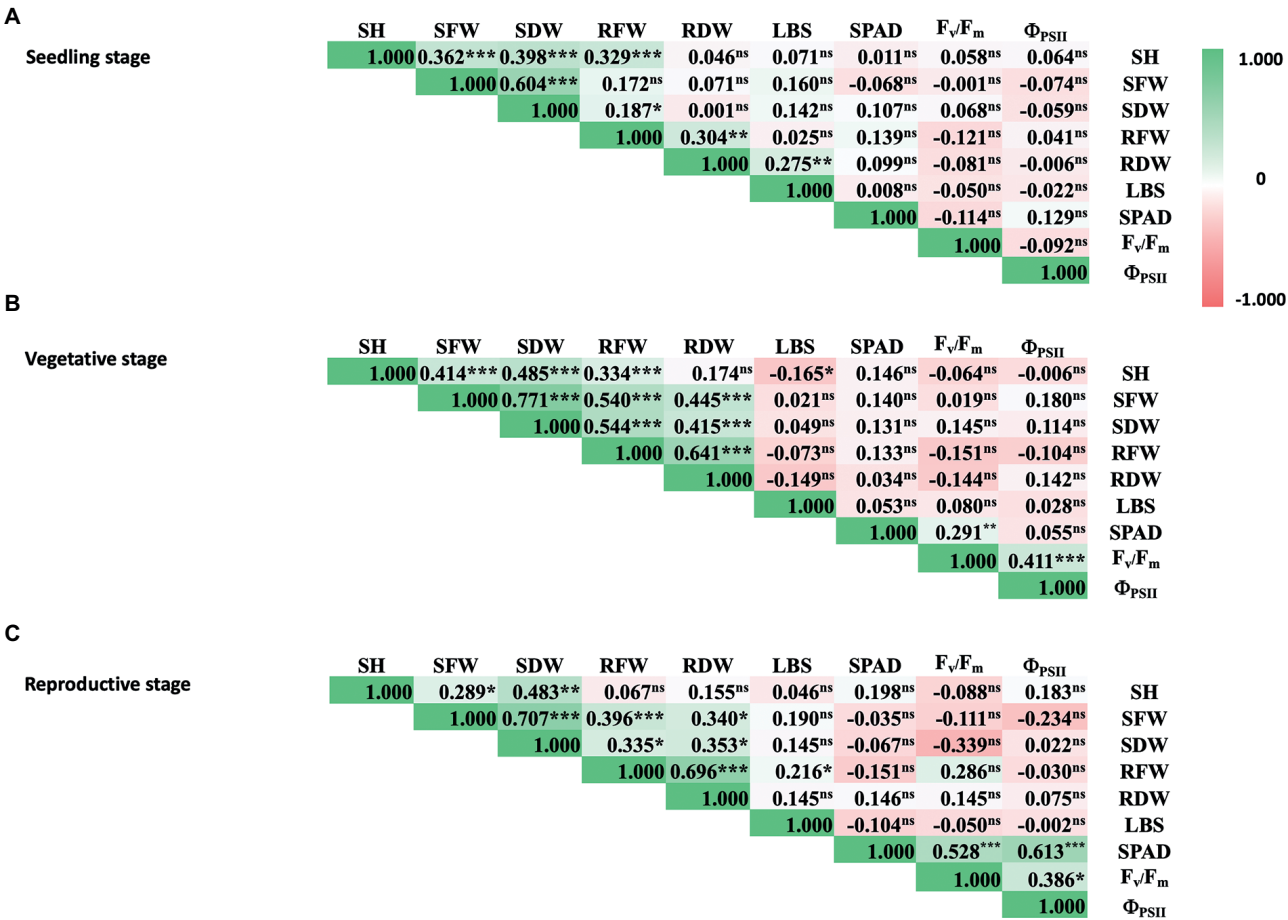
Correlation matrix of physiological and agro-morphological traits of rice at seedling **(A)**, vegetative **(B)**, and reproductive stages **(C)** under 100 mM FeSO_4_ conditions. Colored bars represent correlation values from −1.0 (red) to 1.0 (green). SH, shoot height; SFW, shoot fresh weight; SDW, shoot dry weight; RFW, root fresh weight; RDW, root dry weight; LBS, leaf bronzing symptom; SPAD, leaf greenness index; F_v_/F_m_, maximum quantum yield of PSII; Φ_PSII_, photon yield of PSII. Pearson correlation coefficients are indicated in the boxes and *p*-values are indicated as 0.001 (***), 0.01 (**), 0.05 (*), or not significant (^ns^).

Further analysis of phenotypic data of rice under iron toxicity showed positive correlation among shoot traits, i.e., SH, SFW, and SDW at seedling stage (Figure 2A). Moreover, pronounced correlations were observed between shoot and root traits at vegetative and reproductive stages, i.e., SH, SFW, SDW, RFW, and RDW (Figures 2B,C). The positive correlation between physiological parameters was also shown for SPAD value, F_v_/F_m_, and Φ_PSII_ at vegetative and reproductive stages. For overall parameters, physiological traits did not correlate with agro-morphological traits at all rice stages, as expected.

### Analyses of linkage disequilibrium, population structure, and individual relatedness

**Figure 3:**
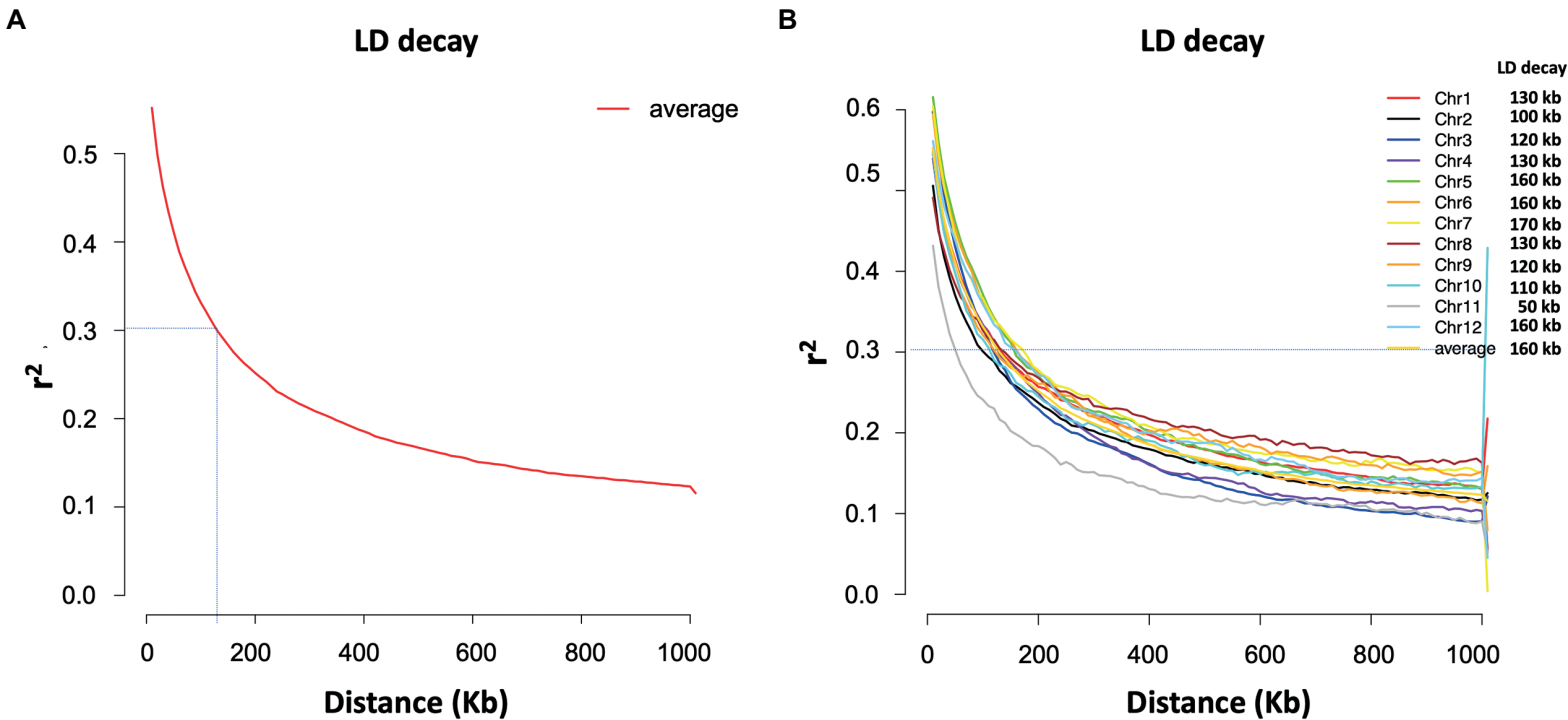
LD decay estimated for the total population (239 accessions). **(A)** Average LD decay. **(B)** Chromosome-wise LD decay.

**Figure 4:**
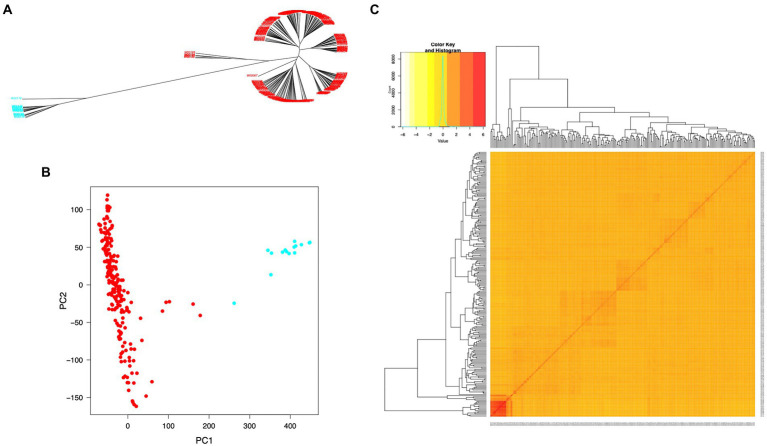
Population structure and individual relatedness of 239 rice accessions. **(A)** Neighbor-joining tree (NJ-tree). **(B)** Two-dimensional plots of PC1 versus PC2. Red and blue colors in **(A)** and **(B)** denote rice accessions in indica and japonica groups, respectively. **(C)** Heatmap of the hierarchical kinship matrix. Relatedness values are represented by color codes in yellow (the highest) and red (the lowest).

The SNP data used in this study included 160,498 SNPs with a minor allele frequency (MAF) of at least 0.05 and a missing genotyping rate of less than 30%. We used all SNPs to calculate pairwise linkage disequilibrium (LD) with PopLDdecay to determine the LD decay rate across all 12 chromosomes. The LD decay distance (*r*^2^ = 0.3) varied from 50 kb (chromosome 11) to 170 kb (chromosome 7), with an average of 160 kb (Figure 3). A neighbor-joining tree (NJ-tree) and PCA analysis were used to evaluate the population structure of 239 rice accessions using the same set of SNPs. Both the NJ-tree and PCA revealed two subpopulations corresponding to the indica and japonica categories (Figures 4A,B). To measure genetic relatedness within the population, a kinship matrix was also constructed. Most accessions had a lower coefficient of relatedness, as shown in the kinship heatmap created for display (Figure 4C).

### GWAS analysis of traits responding to iron toxicity at seedling, vegetative, and reproductive stages

**Figure 5:**
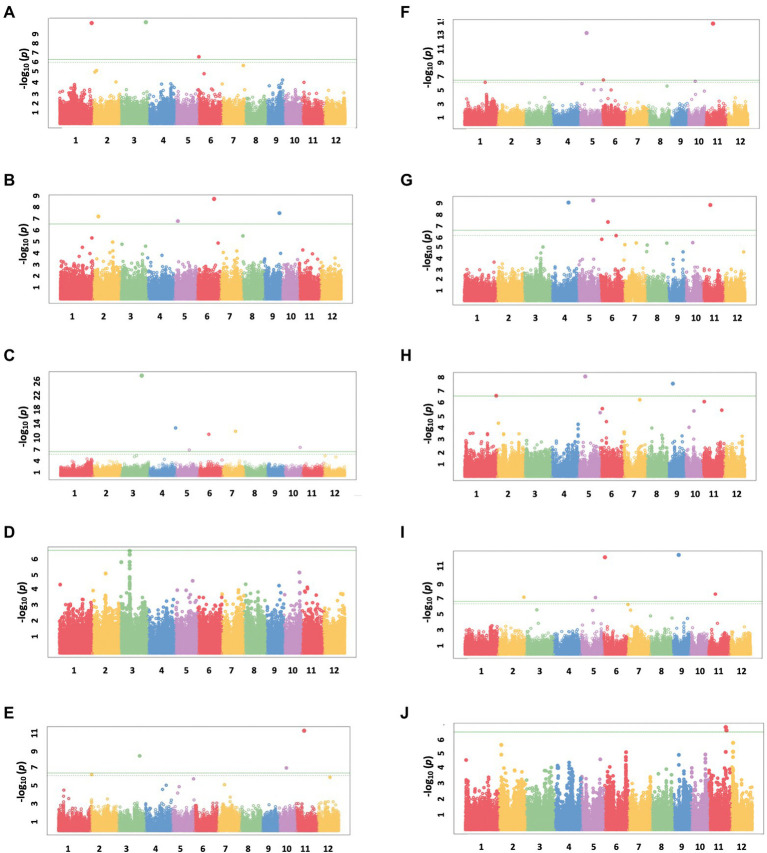
Manhattan plots from FarmCPU results. **(A)** SH at the seedling stage, **(B)** SFW at the seedling stage, **(C)** SDW at the seedling stage, **(D)** F_v_/F_m_ at the seedling stage, **(E)** SFW at the vegetative stage, **(F)** SDW at the vegetative stage, **(G)** RFW at the vegetative stage, **(H)** RDW at the vegetative stage, **(I)** LBS at the vegetative stage, and **(J)** F_v_/F_m_ at the vegetative stage. The chromosomal SNPs are differentiated by various colors. The Bonferroni-corrected *p* = 0.05 and the FDR-corrected *p* = 0.1 are delineated with a horizontal green line and dashed line, respectively.

**Figure 6:**
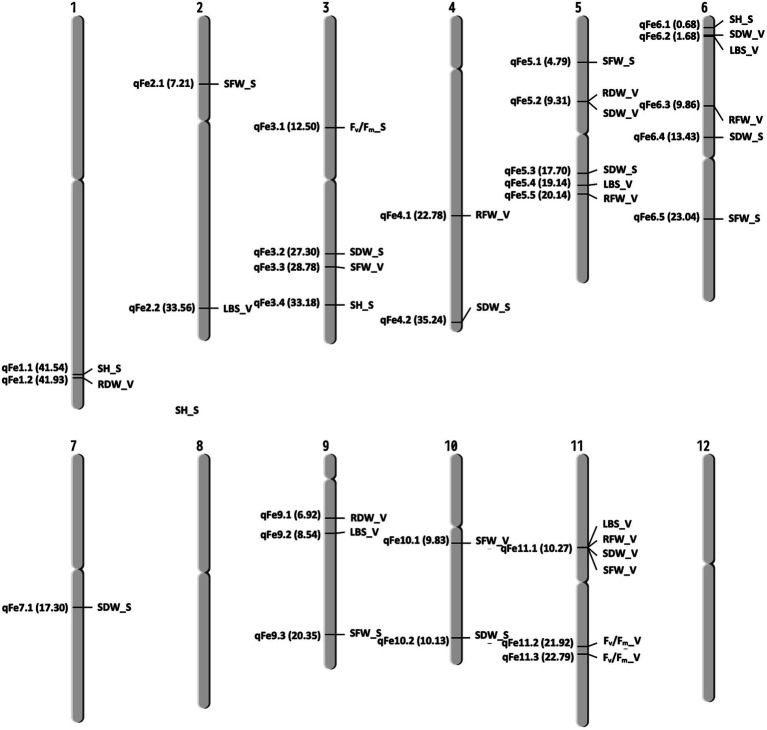
Chromosomal location of QTLs associated with different traits at seedling and vegetative stages. S, seedling stage; V, vegetative stage.

A total of 13 traits were selected to perform GWAS based on trait differences (*p* ≤ 0.01) between control and iron toxicity conditions (Figure 1). These included six traits from the seedling stage, i.e., SH, SFW, SDW, RFW, LBS, and F_v_/F_m_, six traits from the vegetative stage, i.e., SFW, SDW, RFW, LBS, SPAD value, and F_v_/F_m_, and one trait from the reproductive stage, i.e., LBS. Based on the FarmCPU results, 34 significant SNPs (−log_10_*P* > 6.50) were found to be associated with the 12 traits (Figure 5; Table 1). Of these, 14 significant SNPs were associated with the four traits at the seedling stage, i.e., F_v_/F_m_, SDW, SFW, and SH, and 20 significant SNPs were associated with the six traits at the vegetative stage, i.e., F_v_/F_m_, LBS, RDW, RFW, SDW, and SFW (Figure 5; Table 1). At the reproductive stage, no significant SNP was found associated with the trait LBS. In summary, a total of 29 QTLs were identified on ten chromosomes (Figure 6; Table 2). Among them, three common QTLs were detected, namely *qFe5.2* on chromosome 5 for RDW and SDW at the vegetative stage, *qFe6.2* on chromosome 6 for LBS and SDW at the vegetative stage, and *qFe11.1* on chromosome 11 associated with LBS, RFW, SDW, and SFW at the vegetative stage (Figure 6; Table 2). Several QTLs identified in this study were overlapped with previously identified QTLs from bi-parental QTL mapping and GWAS experiments (Table 2).

**Table 1:** Associated SNPs for various traits determined at seedling and vegetative stages.

Stage	Trait	Significant SNPs	Chromosome	Position	−log10(*p*)	No. of samples	MAF
**Seedling**	F_v_/F_m_	R03012626756	3	12,626,756	6.46	166	0.49
	SDW	R03027421097	3	27,421,097	26.97	166	0.08
	SDW	R04035408074	4	35,408,074	12.88	166	0.09
	SDW	R05017867925	5	17,867,925	6.95	166	0.11
	SDW	R06013599134	6	13,599,134	11.18	166	0.05
	SDW	R07017473450	7	17,473,450	11.99	166	0.07
	SDW	R10021099751	10	21,099,751	7.64	166	0.13
	SFW	R02007391064	2	7,391,064	7.16	166	0.23
	SFW	R05004952006	5	4,952,006	6.76	166	0.33
	SFW	R06023203138	6	23,203,138	8.68	166	0.30
	SFW	R09020527426	9	20,527,426	7.45	166	0.22
	SH	R01041710495	1	41,710,495	10.19	166	0.42
	SH	R03033307612	3	33,307,612	10.27	166	0.22
	SH	R06000844379	6	844,379	6.77	166	0.27
**Vegetative**	F_v_/F_m_	R11022061597	11	22,061,597	6.83	213	0.06
	F_v_/F_m_	R11022936441	11	22,936,441	6.62	213	0.05
	LBS	R02033733683	2	33,733,683	7.02	213	0.43
	LBS	R05019302273	5	19,302,273	6.97	213	0.39
	LBS	R06001827820	6	1,827,820	11.91	213	0.18
	LBS	R09008711616	9	8,711,616	12.2	213	0.26
	LBS	R11010414596	11	10,414,596	7.4	213	0.05
	RDW	R01042108835	1	42,108,835	6.53	213	0.40
	RDW	R05009479023	5	9,479,023	8.08	213	0.07
	RDW	R09006928295	9	6,928,295	7.5	213	0.32
	RFW	R04022947099	4	22,947,099	9.07	213	0.06
	RFW	R05020308337	5	20,308,337	9.27	213	0.05
	RFW	R06010026825	6	10,026,825	7.27	213	0.10
	RFW	R11010450719	11	10,450,719	8.84	213	0.06
	SDW	R05009479023	5	9,479,023	13.4	213	0.07
	SDW	R06001777372	6	1,777,372	6.55	213	0.38
	SDW	R11010450719	11	10,450,719	14.77	213	0.06
	SFW	R03028904055	3	28,904,055	8.42	213	0.29
	SFW	R10009982279	10	9,982,279	7.07	213	0.44
	SFW	R11010450719	11	10,450,719	11.25	213	0.06

**Table 2:** Summary of identified QTLs on each chromosome significantly associated with traits.

QTL	Trait	Chr.	Significant SNPs	Flanking region (Mb)	Previously reported QTLs
*qFe1.1*	*SH_S*	1	41,710,495	41.54–41.88	SDW (Dufey et al., 2012)
*qFe1.2*	*RDW_V*	1	42,108,835	41.93–42.27	
*qFe2.1*	*SFW_S*	2	7,391,064	7.21–7.56	
*qFe2.2*	*LBS_V*	2	33,733,683	33.56–33.90	F_v_/F_m_ (Dufey et al., 2012)
*qFe3.1*	*F_v_/F_m__S*	3	12,626,756	12.50–12.74	
*qFe3.2*	*SDW_S*	3	27,421,097	27.23–27.54	SDW (Dufey et al., 2012)
*qFe3.3*	*SFW_V*	3	28,904,055	28.78–29.02	Leaf bronzing index (Dufey et al., 2012)
*qFe3.4*	*SH_S*	3	33,307,612	33.18–33.42	
*qFe4.1*	*RFW_V*	4	22,947,099	22.78–23.16	Harvest index (Diop et al., 2020)
*qFe4.3*	*SDW_S*	4	35,408,074	35.24–35.56	
*qFe5.1*	*SFW_S*	5	4,952,006	4.79–5.11	
qFe5.2	*RDW_V, SDW_V*	5	9,479,023	9.31–9.63	SPAD ratio (Kaewcheenchai et al., 2021); SDW, RDW (Dufey et al., 2015)
*qFe5.3*	*SDW_S*	5	17,867,925	17.70–18.02	
*qFe5.4*	*LBS_V*	5	19,302,273	19.14–19.46	Fe in root-plaque system (Dufey et al., 2015)
*qFe5.5*	*RFW_V*	5	20,308,337	20.14–20.46	Fe in root-plaque system (Dufey et al., 2015)
*qFe6.1*	*SH_S*	6	844,379	0.68–1.00	
*qFe6.2*	*LBS_V, SDW_V*	6	1,777,372	1.61–1.93	
*qFe6.3*	*RFW_V*	6	10,026,825	9.86–10.18	SFW, SDW (Zhang et al., 2017); SFW, SDW (Dufey et al., 2015)
*qFe6.4*	*SDW_S*	6	13,599,134	13.43–13.75	LBS (Matthus et al., 2015)
*qFe6.5*	*SFW_S*	6	23,203,138	23.04–23.36	
*qFe7.1*	*SDW_S*	7	17,473,450	17.30–17.64	Leaf bronzing score (Dufey et al., 2015)
*qFe9.1*	*RDW_V*	9	6,928,295	6.80–7.04	
*qFe9.2*	*LBS_V*	9	8,711,616	8.54–8.88	
*qFe9.3*	*SFW_S*	9	20,527,426	20.35–20.69	SDW (Matthus et al., 2015)
*qFe10.1*	*SFW_V*	10	9,982,279	9.83–10.13	
*qFe10.2*	*SDW_S*	10	21,099,751	21.03–21.40	Leaf bronzing index, 100-grain weight (Dufey et al., 2012)
*qFe11.1*	*LBS_V, RFW_V, SDW_V, SFW_V*	11	10,450,719	10.27–10.55	SDW (Dufey et al., 2012)
*qFe11.2*	*F_v_/F_m__V*	11	22,061,597	21.92–22.20	Shoot water content (Dufey et al., 2012)
*qFe11.3*	*F_v_/F_m__V*	11	22,936,441	22.79–23.07	SFW, SDW, RDW (Zhang et al., 2017); Relative root length (Matthus et al., 2015) SFW, SDW, RDW, relative root length (Dufey et al., 2012)

S, seedling stage; V, vegetative stage.

### Analysis of candidate genes

**Figure 7:**
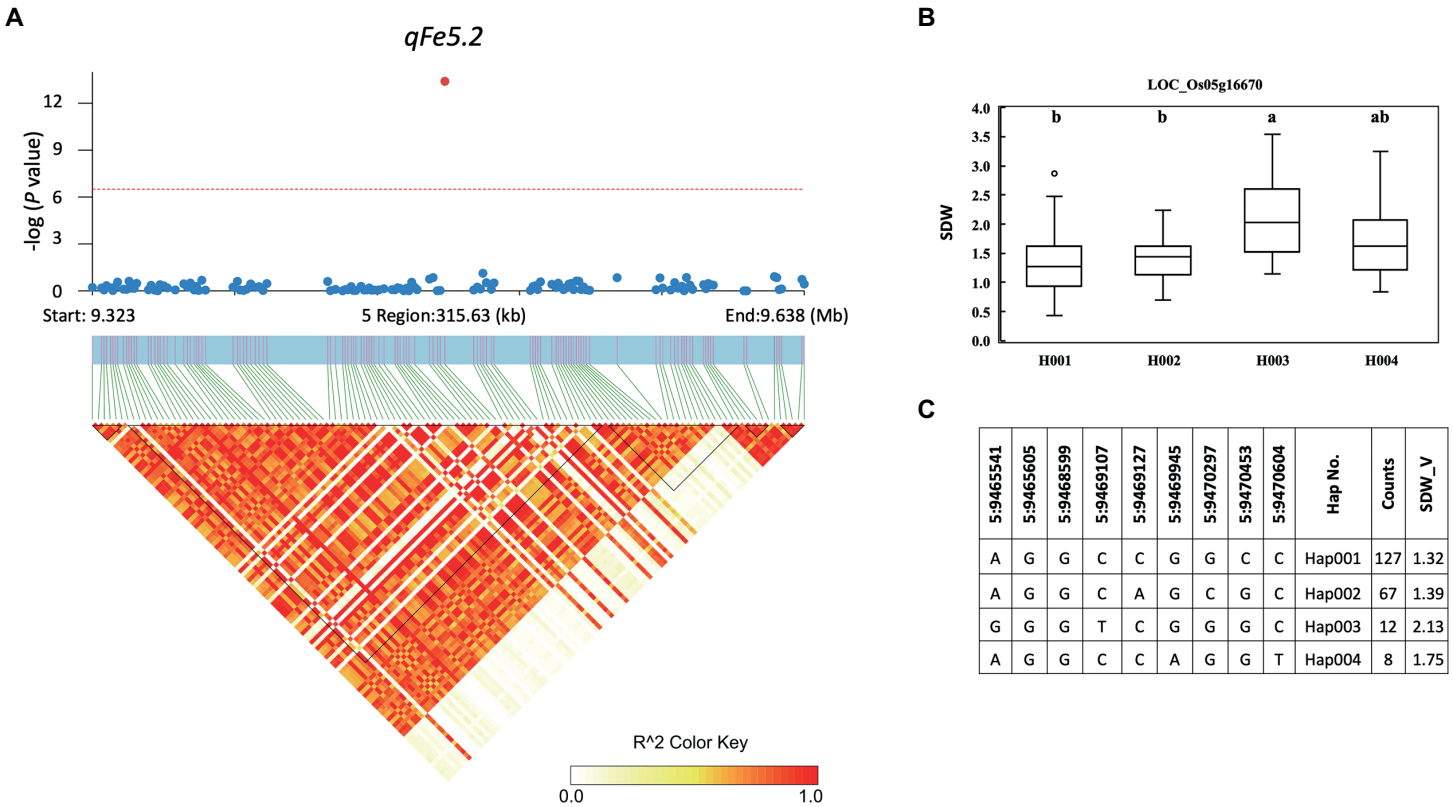
Associated regions and haplotype analysis of *qFe5.2*. **(A)** Regional Manhattan plots and LD heatmap across the 315-kb region surrounding the significant SNP. The single red dot above the red line denotes the significant SNP. The black triangles in the LD heatmap represent the haploblocks. **(B)** Boxplots show the phenotypic distribution of SDW in the LOC_Os005g16670 haplotype groups. Different letters above each haplotype group show significant difference at p ≤ 0.05 according to Tukey’s HSD test. **(C)** Haplotype analysis of LOC_Os005g16670.

**Figure 8:**
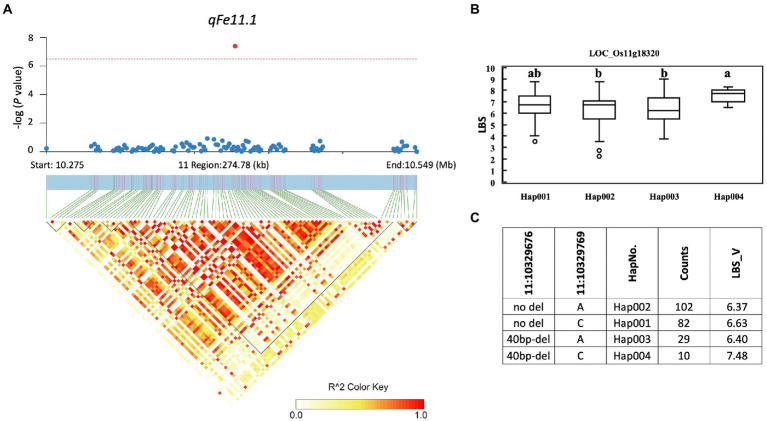
Associated regions and haplotype analysis of *qFe11.1*. **(A)** Regional Manhattan plots and LD heatmap across the 275-kb region surrounding the significant SNP. The single red dot above the red line denotes the significant SNP. The black triangles in the LD heatmap represent the haploblocks. **(B)** Boxplots show the phenotypic distribution of LBS in the LOC_Os0011g18320 haplotype groups. Different letters above each haplotype group show significant difference at p ≤ 0.05 according to Tukey’s HSD test. **(C)** Haplotype analysis of LOC_Os005g18320.

A total of 857 genes were identified in the interval of ± LD decay in each chromosome (Supplementary Table S3). Among these genes, based on the Rice Genome Annotation Project^1^ annotations, 44 genes were annotated with the gene ontology (GO) “response to abiotic stimulus” and 22 genes were annotated with the GO “transporter activity” (Supplementary Table S4). Two genes previously reported to be associated with iron homeostasis were also identified, namely LOC_Os01g72370 (*OsIRO2, OsbHLH056*) and LOC_Os04g38570 (multidrug resistance protein: *OsABCB14*), which were reported to play a role in iron homeostasis in rice (Ogo et al., 2007; Xu et al., 2014). Among all the identified QTLs, we further examined the regions colocalized with two or more traits and overlapped with those previously reported. These included the QTLs *qFe5.2* on chromosome 5 and *qFe11.1* on chromosome 11. For *qFe5.2*, a total of 16 genes were subjected to gene-based haplotype analysis with RDW and SDW at the vegetative stage (Supplementary Table S3). Of these genes, only LOC_Os05g16670 (SHR5-receptor-like kinase) were found to contain different haplotypes that were associated with the difference in SDW. Among the four haplotypes, Hap003 was significantly associated with higher SDW, while Hap001 and Hap002 were significantly associated with lower SDW (Figure 7). However, these haplotypes were not significantly associated with the difference in RDW (data not shown). For *qFe11.1*, 13 genes were subjected to gene-based haplotype analysis with LBS, FRW, SFW, and SDW at the vegetative stage. The result was that four genes, namely LOC_Os11g18320 (expressed protein), LOC_Os11g18366 (cycloartenol synthase), LOC_Os11g18570 (cytochrome P450), and LOC_Os11g18660 (expressed protein), were found to contain different haplotypes. Among these, only LOC_Os11g18320 had haplotypes that were significantly associated with the difference in LBS. Among the four haplotypes, Hap004 was significantly associated with higher LBS, while Hap002 and Hap003 were associated with lower LBS (Figure 8). However, these haplotypes were not significantly associated with RFW, SFW and SDW at the vegetative stage (data not shown).

## Discussion

In the present study, the differences in agro-morphological traits such as shoot height (SH), shoot fresh weight (SFW), shoot dry weight (SDW), root fresh weight (RFW) and root dry weight (RDW) and in physiological traits such as maximum quantum yield (F_v_/F_m_), photon yield of PSII (Φ_PSII_), leaf greenness, and leaf bronzing value (LBS) between Fe toxicity (Fe + pH 4.5) and the control (pH 4.5) at seedling, vegetative, and reproductive stages were compared in a diversity panel of 239 accessions. Of these traits, LBS showed the greatest differences at all stages. In particular, at the seedling stage, the largest median difference of 65.2% was found from LBS. LBS was identified as the major symptom and the most recognized morphological changes when rice was exposed to Fe toxicity. Previous reports mentioned LBS as a trait to identify candidate genes for Fe toxicity tolerance (Wu et al., 2014; Matthus et al., 2015; Diop et al., 2020). Another trait that showed a large difference at the seedling stage is SDW with a median difference of 40%. However, this trait showed little difference at the vegetative stage and no significance at the reproductive stage. SDW was previously used as a parameter to identify QTLs associated with Fe toxicity tolerance at the seedling stage (Kaewcheenchai et al., 2021). For the other traits, the median difference is less than 30% at all stages. According to these results, LBS is suggested as a key parameter for determining Fe toxicity response at all stages, and SDW could be a promising parameter at the seedling stage. In addition, our results showed a significant reduction in morphological and physiological traits at the seedling and vegetative stages in Fe toxicity, whereas only minor phenotypic changes were observed at the reproductive stage.

Several studies have mapped the rice genome in the context of traits related to Fe toxicity tolerance under different environmental conditions and using different segregating populations from intraspecific populations or interspecific populations (Wan et al., 2003; Ouyang et al., 2007; Shimizu, 2009; Dufey et al., 2015; Diop et al., 2020). To identify QTLs associated with these evaluated traits, we performed GWAS using the FarmCPU model in GAPIT version 3. FarmCPU is a multilocus model that has been shown to be more robust in controlling for false positives and false negatives (Kaler et al., 2019; Kumar et al., 2022). This was also evident in the Q-Q plots (Supplementary Figure S3). We looked at all traits that showed a significant median difference in comparison between Fe toxicity and control to identify QTLs associated with each trait, as well as the colocalized QTLs of these traits. Although LBS had the highest trait difference between Fe toxicity and control at all stages, significant QTLs associated with the trait were identified only at the vegetative stage. This could be due to the differences in samples available to perform GWAS at each stage and the quality of the data. Among the 29 QTLs identified in this study, we found three that were associated with more than two traits at the vegetative stage. In particular, *qFe11.1* proved to be a colocalized QTL for four traits, i.e., LBS, RFW, SDW, and SFW. With the exception of LBS, these traits showed a high correlation with each other (Figure 2). Interestingly, 16 of the 29 QTLs identified in this study were found to be colocalized or overlapping with previously reported QTLs identified by traditional QTL mapping and association mapping (Table 2).

Although a large number of genes were annotated for all identified QTLs, only a few genes associated with Fe toxicity were identified. These include LOC_Os01g72370 (*OsIRO2, OsbHLH056*) and LOC_Os04g38570 (multidrug resistance protein: *OsABCB14*). In a previous study, *OsIRO2* was found to be downregulated under Fe toxicity stress, which likely serves to prevent Fe toxicity (Turhadi et al., 2019). *OsABCB14* has been described as an auxin transporter affecting iron homeostasis in rice (Xu et al., 2014). Furthermore, we performed gene-based haplotype analysis to prioritize the candidate genes annotated within the two colocalized QTLs *qFe5.2* and *qFe11.1*, which overlap with other previously reported QTLs. *qFe5.2* was found to be a colocalized QTL associated with RDW and SDW at the vegetative stage. This QTL was also found to overlap with QTLs associated with SPAD ratio (Kaewcheenchai et al., 2021) and SDW and RDW (Dufey et al., 2015). Of the 16 genes annotated for *qFe5.2*, only the gene LOC_Os05g16670 was found to contain different haplotypes based on nonsynonymous SNPs of this gene. Although RDW and SDW were associated with this QTL at the vegetative stage, only the difference in SDW was associated with haplotypes of this gene. LOC_Os05g16670 encodes SHR5-receptor-like kinase. This gene in sugarcane has been reported as a plant receptor kinase involved in the association of plant-N_2_-fixing endophytic bacteria (Vinagre et al., 2006). The function of this gene in relation to Fe toxicity in rice has not been reported. *qFe11.1* was also found to be colocalized QTL associated with four traits, i.e., LBS, RFW, SDW, and SFW at the vegetative stage. This QTL overlaps with the previously reported QTL for SDW (Dufey et al., 2012). Of 13 genes identified within this QTL, four genes contained haplotypes. However, only the gene LOC_Os11g18320 had haplotypes associated with the difference in LBS. Unfortunately, LOC_Os11g18320 has no annotated function according to the Rice Genome Annotation Project annotation. Because this study identified many QTLs for traits associated with Fe toxicity responses and several hundred genes are involved, their use in breeding programs is still difficult. Further study is needed to verify the functions of the proposed candidate genes and other genes in important QTLs before using them in breeding programs for Fe toxicity tolerance.

## Conclusion

In conclusion, among agro-morphological and physiological parameters, LBS is a key parameter for determining Fe toxicity response at all stages, and SDW could be a promising parameter at the seedling stage. This study also provides an insight into the QTL locations and candidate genes associated with traits that respond to Fe toxicity at different stages. A total of 29 QTLs associated with agro-morphological and physiological traits responsive to Fe toxicity at seedling and vegetative stages were identified. Among them, three colocalized QTLs associated with more than two traits were identified on chromosomes 5, 6, and 11. Based on functional annotations, two genes previously reported to be associated with Fe toxicity were identified. Two additional genes were identified as promising candidates for the colocalized QTLs on chromosomes 5 and 11.

## Data availability statement

The SNP dataset presented in this study can be found in Zenodo repository (https://doi.org/10.5281/zenodo.7110300).

## Author contributions

TT: project leader, experimental layout, and manuscript preparation. SC-u: data analysis, experimental layout, and manuscript preparation. CT: data analysis, manuscript preparation, phenotypic data collection, and execution of experiment. SW: data analysis, manuscript preparation, genotypic data collection, and execution of experiment. VR, RT, TSa, and TSo: measurement of morphological traits in seedling, vegetative and reproductive stages, and execution of experiment. All authors contributed to the article and approved the submitted version.

## Funding

This work was supported by the National Science and Technology Development Agency (NSTDA; grant number P-18-51456).

## Conflict of interest

The authors declare that the research was conducted in the absence of any commercial or financial relationships that could be construed as a potential conflict of interest.

## Publisher’s note

All claims expressed in this article are solely those of the authors and do not necessarily represent those of their affiliated organizations, or those of the publisher, the editors and the reviewers. Any product that may be evaluated in this article, or claim that may be made by its manufacturer, is not guaranteed or endorsed by the publisher.

## Footnotes

^1^ http://rice.uga.edu
