# Supplementary material for: Determination of traits responding to iron toxicity stress at different stages and genome-wide association analysis for iron toxicity tolerance in rice (Oryza sativa L.)
Source: Front Plant Sci. 2022 Oct 6;13:994560. doi: 10.3389/fpls.2022.994560 (PMC9583542; doi:10.3389/fpls.2022.994560)
Supplement: Supplementary file 1 [file Data_Sheet_1.zip › Figure S3.DOCX]

**Figure S3.** Quantile-quantile (Q-Q) plots from FarmCPU results. (A.) SH at the seedling stage, (B.) SFW at the seedling stage, (C.) SDW at the seedling stage, (D.) Fv/Fm at the seedling stage, (E.) SFW at the vegetative stage, (F.) SDW at the vegetative stage, (G.) RFW at the vegetative stage, (H.) RDW at the vegetative stage, (I.) LBS at the vegetative stage, and (J.) Fv/Fm at the vegetative stage.
