# Supplementary figures and images for: Determination of traits responding to iron toxicity stress at different stages and genome-wide association analysis for iron toxicity tolerance in rice (Oryza sativa L.)
Source: Front Plant Sci. 2022 Oct 6;13:994560. doi: 10.3389/fpls.2022.994560 (PMC9583542; doi:10.3389/fpls.2022.994560)

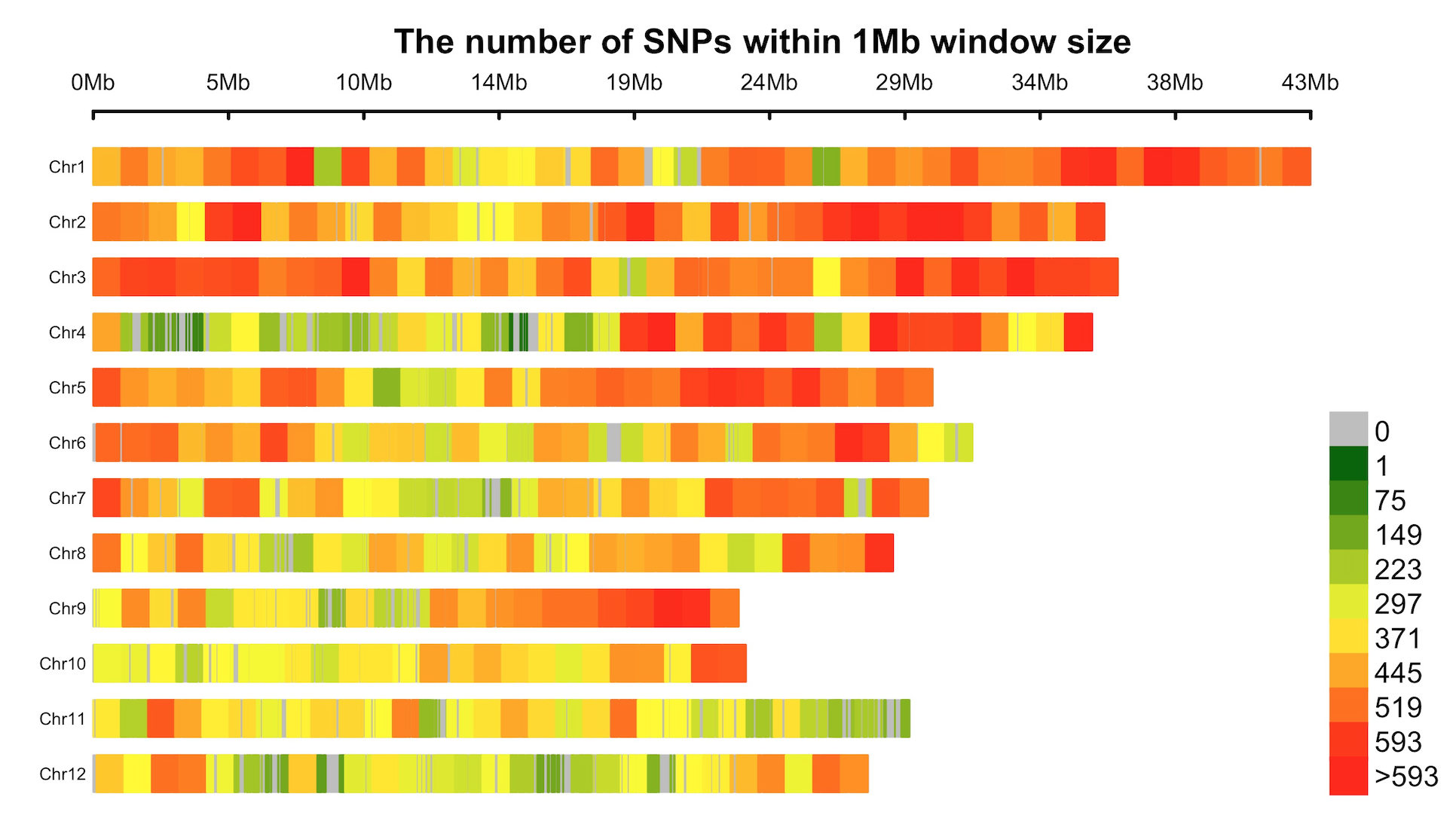


Figure S1. Density heatmap of the 160,498 SNP markers across 12 rice chromsome

Supplement: Supplementary file 1 [file Data_Sheet_1.zip › Figure S1.DOCX]

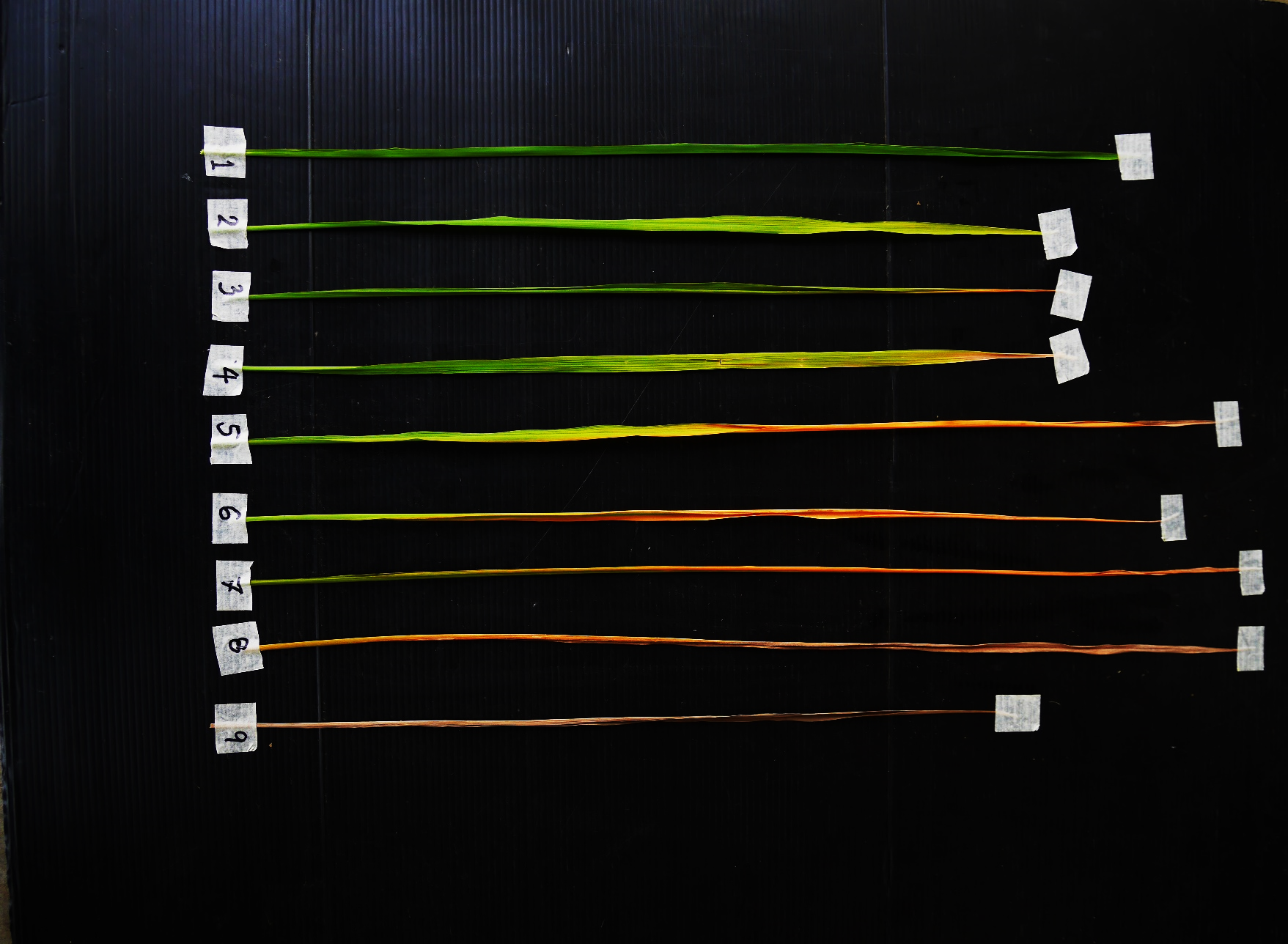


**Figure S2.** Visual leaf bronzing scores from 1 to 9.

Supplement: Supplementary file 1 [file Data_Sheet_1.zip › Figure S2.DOCX]
